# Urea assisted ceria nanocubes for efficient removal of malachite green organic dye from aqueous system

**DOI:** 10.1038/s41598-019-50984-6

**Published:** 2019-10-09

**Authors:** Thupakula Venkata Madhukar Sreekanth, Patnamsetty Chidanandha Nagajyothi, Gutturu Rajasekhara Reddy, Jaesool Shim, Kisoo Yoo

**Affiliations:** 1 School of Mechanical Engineering, Yeungnam University, Gyeongsan, 38541 Republic of Korea; 2 Department of Instrumentation, Sri Venkateswara University, Tirupati, 517 502 India

**Keywords:** Natural hazards, Nanoparticles

## Abstract

This study describes a simple, high-yield, rapid, and inexpensive route for the synthesis of cubic shape-like cerium oxide nanocubes (CeO_2_ NCs) using different urea concentrations (0.5, 1.0, and 2.0 g) by the hydrothermal method. The synthesized nanocubes (NCs) are labeled as CeO_2_ NCs-0.5, CeO_2_ NCs-1.0, and CeO_2_ NCs-2.0, corresponding to 0.5, 1.0, and 2.0 g of urea, respectively. The synthesized NCs were characterized by FT-IR, UV-visible, XRD, XPS, SEM and HR-TEM analysis. The synthesized NCs were cubic in shape with average sizes of 12, 12, and 13 nm for the CeO_2_ NCs-0.5, CeO_2_ NCs-1.0, and CeO_2_ NCs-2.0, respectively, obtained by the XRD analysis. The catalytic activity of the CeO_2_ NCs was studied for the purpose of obtaining the reduction of malachite green (MG) in the presence of sodium borohydride (NaBH_4_) at room temperature.

## Introduction

Environmental pollution is one of the most serious problems facing human beings and other life forms due to the increasing population, industrialization, and urbanization^1^. Dyes are major pollutants that are released from textile industrial effluent^2^. Malachite green (MG, including aniline green; basic green 4; diamond green B; and victoria green B) is a water-soluble cationic dye that is available in two forms malachite green chloride (C_23_H_25_ClN_2_) and malachite green oxalate (C_52_H_54_N_4_O_12_) (Table 1). It is used as an industrial dye for the dyeing of leather, wool, silk, cotton, jute, paper and the manufacturing of printing inks and paints^3^. The extensive usage of MG dye has caused several health problems, including significant effects on the immune and reproductive systems^4^. MG also has the potential to cause genotoxic and carcinogenic effects and is extremely cytotoxic to mammalian cells^5^, and hence the suitable treatment of wastewater containing MG dye is highly necessary. Several methods have been developed, such as physical, chemical, biological, and photocatalytic degradation methods for the treatment of industrial effluent^3,6–9^. The physical and chemical methods include adsorption^10^, ion-exchange^11^, irradiation^12^, oxidation processing^13^, chemical precipitation^14^, photolysis^15^, coagulation/flocculation^16^, electrochemical treatment^17^, ozonisation^18^, photo-Fenton degradation^19,20^, reduction and oxidation^21^. The biological methods include fungal degradation^22^, bacterial degradation^23^, and aerobic and anaerobic degradation^24^. However, these methods are generally expensive, are unable to completely remove the dye, and produce high sludge and by-products that cause secondary pollution^25^. In recent years photocatalytic degradation of organic dyes has been used effectively, but this process is slow and energy-consuming. Compared with photocatalytic activity, a catalytic reduction is a relatively fast process without high energy requirements^26^.

**Table 1:** Chemical properties of malachite green (MG) dye.

Properties	Malachite green chloride	Malachite green oxalate
Molecular formula	C_23_H_25_ClN_2_	C_52_H_54_N_4_O_12_
Molecular weight	364.911 g/mol	927.00 g/mol
Max. wavelength (*λ*_max_)	618 nm	618 nm
Structure		

Ce is a rare earth element with a wide bandgap (~3.2 eV), lanthanide series, and exists as a free metal or oscillates between in the Ce^3+^ and Ce^4+^ oxidation states^27^. CeO_2_ NCs, also known as nanoceria, have been widely used in catalysis, fast-ion conductors, UV blockers, energy storage, and optical sensors due to its excellent physical and chemical properties^28–31^.

CeO_2_ is an effective catalyst for the removal of organic dyes from effluents because ceria also hops between Ce^3+^ and Ce^4+^ valence states containing oxygen vacancies that allow NCs to act as regenerative catalysts^32^. Various methods have been reported in the literature for the synthesis of ceria^33–37^, including sol-gel^38^, microwave combustion^39^, flame spray pyrolysis^40^, solvothermal^41^, microemulsion^42^, spray drying system^43^, hydrothermal^44^, and thermal decomposition^45^. Among these, the hydrothermal method is simple, which can produce high product yields^46^.

In this study we synthesized CeO_2_ NCs using urea (CH_4_N_2_O), as capping and reducing agent, urea was also known as carbamide and it is easily soluble in water. It has been used as a nitrogen source for the synthesis of environmentally benign nanoparticles. It has been widely used in many industries such as in agriculture, plastic, drug, soap, and detergent industries. However, excess concentrations urea in soil or in water causes to soil acidification and eutrophication and toxic effects to aquatic organisms, animals and humans^47,48^. The ceria nanocubes (CeO_2_ NCs) were synthesized via the one-pot hydrothermal method using different concentrations of urea. The CeO_2_ NCs are applied as a catalyst for the degradation of MG in the presence of NaBH_4_ in an aqueous medium using UV-visible spectroscopy.

## Materials and Methods

### Materials

Analytical grade cerium nitrate hexahydrate and urea were purchased from Alfa-Aesar, South Korea and used directly. Malachite green dye was obtained from Daejung Chemicals, South Korea. Deionized (DI) water was used throughout the study for the synthesis of CeO_2_ NCs and dye degradation studies.

### Synthesis of ceria nanocubes

Cerium nitrate hexahydrate, 0.01 M was prepared at a volume of 70 mL in DI water. Different quantities of urea (0.5, 1.0 and 2.0 g) were added and stirred until dissolved. This reaction mixture was transferred to an autoclave at a temperature of 180 °C for 6 h. The reaction solution was naturally cooled down to room temperature. The reaction mixture was carefully collected by centrifuge and wished with DI and ethanol to remove the unreacted substance, before drying at 80 °C overnight. This was later calcined at 400 °C for 4 h and for comparison we prepared ceria without urea (CeO_2_ NCs-0.0).

### Characterization

The crystallinity was measured by an X-ray diffractometer (PANalytical X-Pert PRO, USA) using a Cu Kα source (λ = 1.5405 Å). FT-IR (Perkin-Elmer, Bruker) was used to identify the vibrational modes by an ATR mode. The morphology of the samples was investigated by scanning electron microscopy (SEM-4800, Hitachi) and high-resolution transmission electron microscopy (TEM, Titan G2 ChemiSTM Cs Probe) with a 200 kV field emission gun in high brightness Schottky mode FEG (X-FEG). UV-visible absorption was measured on a Neogen (NEO-D3117). The elemental composition was quantitatively compared using an X-ray photoelectron spectrometer (K-alpha, Thermo Scientific, USA) with Al Kα radiation (1486.6 eV).

### Catalytic activity

The synthesized samples were used to check the catalytic ability using the MG dye. For the catalytic reactions, 2.5 mL of the MG dye, 25 µL of well-dispersed CeO_2_ NCs (2 mg/mL) and 0.2 mL of (0.2 M) freshly prepared NaBH_4_ were added to all the samples. The degradation efficiency was monitored by UV-visible spectroscopy (Neogen, NEO-D3117). The degradation efficiency was calculated as a function of time for *C*_*o*_*/C*_*t*_ and ln *C*_*o*_*/C*_*t*_, where *C*_*o*_ is initial concentration and *C*_*t*_ is the final concentration of the MG dye. Blank control experiments were performed without catalyst (MG + NaBH_4_) or NaBH_4_ (MG + CeO_2_ NCs) to study the catalyst efficiency.

## Results and Discussion

### Characterization

**Figure 1:**
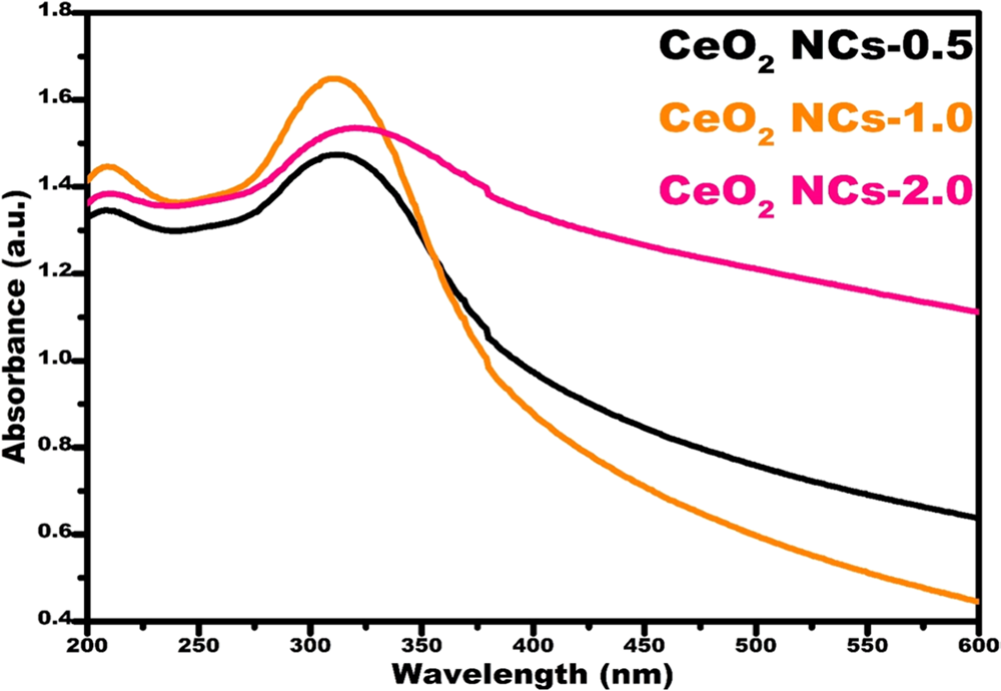
UV-visible spectra of the cerium oxide nanocubes (CeO_2_ NCs) as a function of urea concentration.

The UV-visible absorbance spectra of the ceria nanocubes are shown in Fig. 1. The synthesized ceria nanocubes exhibited broad absorption peaks below 400 nm. Couple of broad absorption peaks were observed at wavelengths 208 and 311 nm (CeO_2_ NCs-0.5); 209 and 310 nm (CeO_2_ NCs-1.0); and 209 and 314 nm (CeO_2_ NCs-2.0). These peaks correspond to the characteristic absorption peaks of Ce^3+^ and Ce^4+^ of the CeO_2_ NCs. These results are similar to those obtained by Nurhasanah^49^ *et al*. who observed absorption peaks at 207 and 303 nm for CeO_2_ NCs (9 nm, precipitation method).

**Figure 2:**
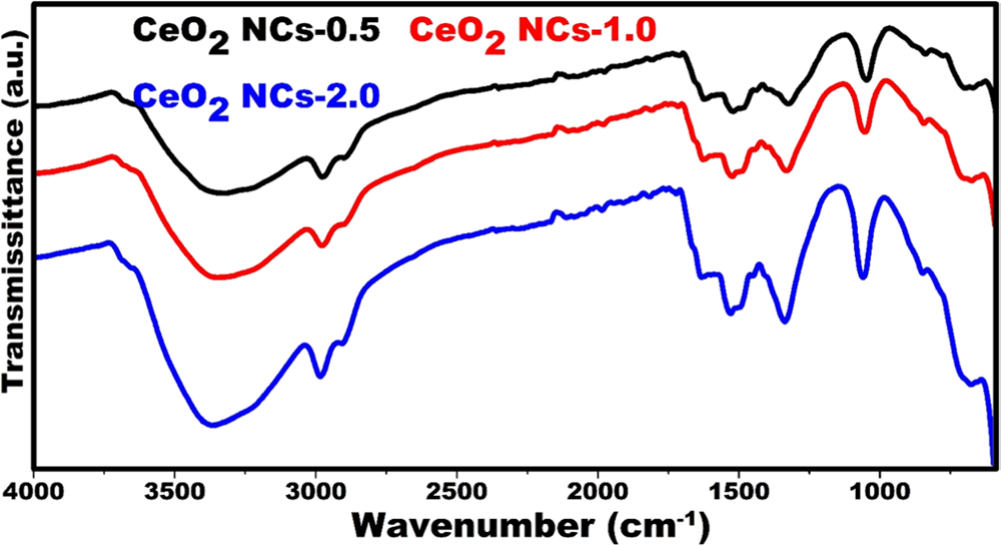
FI-TR spectra of cerium oxide nanocubes (CeO_2_ NCs) as a function of urea concentrations.

Fig. 2 shows the FT-IR spectra of the as-synthesized urea-based ceria nanocubes (CeO_2_ NCs-0.5, CeO_2_ NCs-1.0, and CeO_2_ NCs-2.0). The features near 1540 and 1630 cm^−1^ related to (*ν* C=O) and (N-H stretching) bond stretch^50,51^. The remaining observed peaks are as follows: near 3362 cm^−1^ (O-H stretching vibrations)^52^; near 2980 cm^−1^ (asymmetric C-H stretching)^53^; sharp band near 1340 cm^−1^ (N-O stretch due to the presence of nitrate)^54^; 1071 cm^−1^ (C-O stretching vibration)^55^; 850 cm^−1^ (formed by CeO_2_, which is typical peak of the Ce-O stretching vibrations)^56^; and below 700 cm^−1^ (O-Ce-O stretching vibrations)^57^.

**Figure 3:**
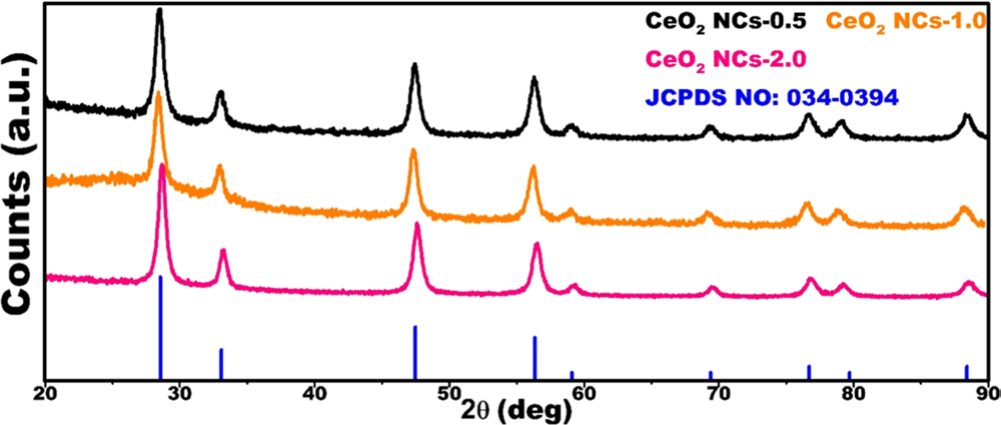
XRD spectra of the cerium oxide nanocubes (CeO_2_ NCs) as a function of urea concentrations.

Fig. 3 shows the XRD patterns of urea-based 0.5, 1.0 and 2.0 CeO_2_ NCs. The XRD revealed sharp peaks which located at 2θ = 28.5°, 32.9°, 47.5°, 56.3°, 59.0°, 69.3°, 76.7°, 78.8°, and 88.4°, are corresponding to (111), (200), (220), (311), (222), (400), (331), (420) and (422) planes, respectively. No extra peaks were observed, which supported the assumption that urea-based NCs are pure with cubic fluorite structure (JCPDC: 034–0394), similar peaks were also observed in CeO_2_ NCs-0.0 and results are shown in Fig. S1. The average mean grain size (MGS) of the NCs was calculated by the Debye- Scherer equation as follows^58,59^:

1 $${\rm{MGS}}=0.94\lambda /\beta cos\theta $$

where *β* is the broadening in the full-width at half maximum (FWHM), *λ* is the X-ray wavelength (1.5406 Å), and *θ* is the Bragg diffraction angle. The microstrain, **ε**, of the NCs is evaluated by

2 $$\varepsilon =(\beta \,\cos \,\theta )/4$$

The dislocation density *δ*, of the NCs is defined as the length of dislocations lines per unit volume and given by

3 $$\delta =1/{(MGS)}^{2}$$

The lattice strain (LS) of the NCs was calculated using the following relation^60,61^.

4 $$LS=\beta /4\,\tan \,\theta $$

From Table 2, the MGS increased with the increasing urea concentration, for CeO_2_ NCs-0.5 and CeO_2_ NCs-1.0 were showing similar value (12 nm), and for CeO_2_ NCs-2.0 showing slightly bigger size (13 nm), whereas CeO_2_ NCs-0.0 showing much higher than (31 nm) urea mediated CeO_2_ NCs, this MGS analysis clearly indicating the urea was acted as capping and reducing agent to control the size of the NCs, meanwhile, the dislocation density decreased with increasing urea concentration, but for higher concentrations (CeO_2_ NCs-1.0 and CeO_2_ NCs-2.0) dislocation density was constant, in CeO_2_ NCs-0.0 too low than the urea mediated CeO_2_ NCs. The microstrain was constant with increasing urea concertation but without urea CeO_2_ NCs showing lower than other CeO_2_ NCs, however, lattice strain (LS) was constant for the first two concentrations (CeO_2_ NCs-0.5 and CeO_2_ NCs-1.0), and for the higher concertation (CeO_2_ NCs-2.0), it was slightly lowered compared with those of the lower concentrations, whereas in CeO_2_ NCs-0.0 showing very lower values, based on these results urea playing vital role as capping and reducing agent in the formation of CeO_2_ NCs.

**Table 2:** Calculated XRD parameters of the urea assisted CeO_2_ NCs.

Sample	Peak degree (°)	Diffraction plane	Mean grain size (nm)	Dislocation density (δ m^−2^)	Microstrain (ε)	Lattice strain (LS)
CeO_2_ NCs-0.5	28.5	111	12	0.006	0.003	0.011
CeO_2_ NCs-1.0	28.5	111	12	0.005	0.003	0.011
CeO_2_ NCs-2.0	28.5	111	13	0.005	0.003	0.010
CeO_2_ NCs-0.0	28.3	111	31	0.0009	0.001	0.004

**Figure 4:**
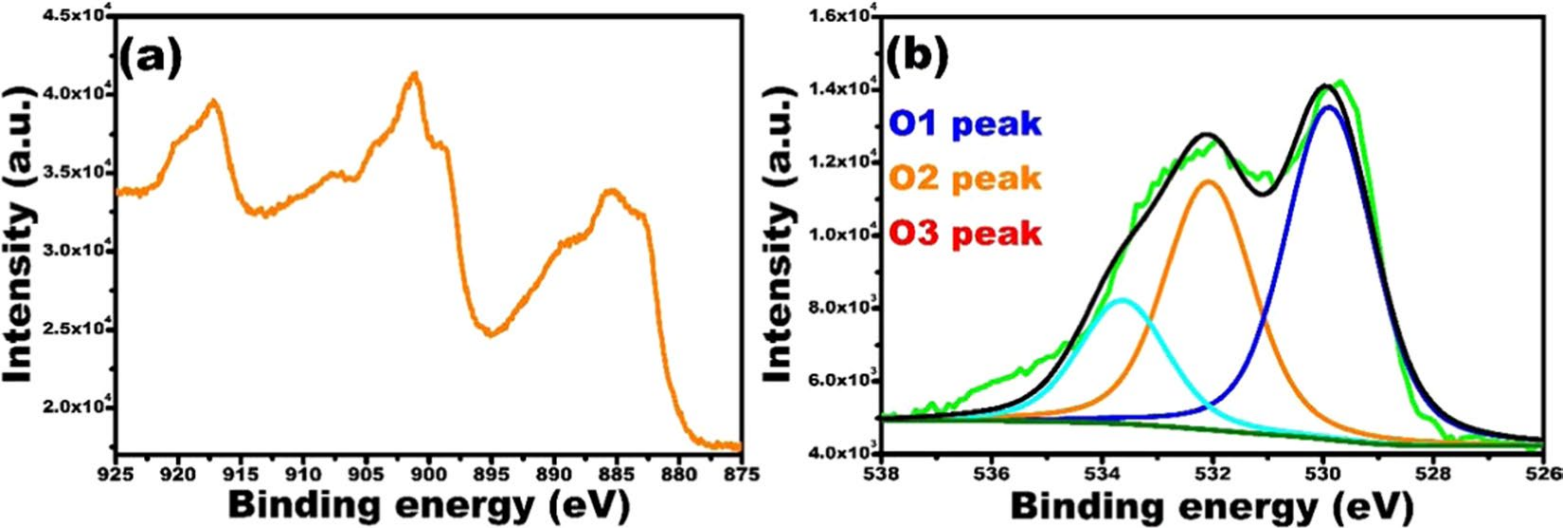
XPS analysis of the CeO_2_ NCs-0.5; high-resolution spectra of cerium (Ce 3d (**a**)) and oxygen (O 1 s (**b**)).

To identify the chemical composition on the surface of the CeO_2_ NCs, we performed the XPS analysis (XPS analysis only for CeO_2_ NCs-0.5) and the results are presented in Fig. 4. The survey scan (Fig. S2(a)) clearly shows the presence of Ce and O elements. The high-resolution XPS spectrum of Ce shown in Fig. 4a contains six peaks, revealing that the Ce contained both the Ce^3+^ and Ce^4+^ oxidation states. The binding energies (BE) at 882.9, 901.2 and 917.2 eV are attributed to Ce^4+^, and the peaks at 885.5, 899.1 and 908.3 eV are the characteristic peaks of Ce^3+^. These results suggest the presence of mixed oxidations and a valence state for the Ce ions in the NCs^62,63^ and Deshpande^64^ *et al*. reported that Ce^3+^ ions create oxygen vacancies in the CeO_2_ lattice that are defects. There were three peaks in the high-resolution O 1 s deconvoluted spectrum in Fig. 4b; the O1 peak, O2 peak, and O3 peak, with 529.8, 532.0, and 533.6 eV binding energies, respectively. The O1 peak (529.8 eV) corresponds to the bond Ce-O, O2 peak (532.0 eV) is attributed to the oxygen vacancies, and O3 peak (533.6 eV) belongs to organic C=O bond or surface –OH groups. To get more information we performed XPS analysis for N 1 s, but the results are not showing any significant peak for N 1 s and results are shown in Fig. S2(b). These results are indicating the urea was acting as capping and reduction the formation of CeO_2_ NCs, not doped in CeO_2_ NCs, which are similar to the values in literature^62,65–67^.

**Figure 5:**
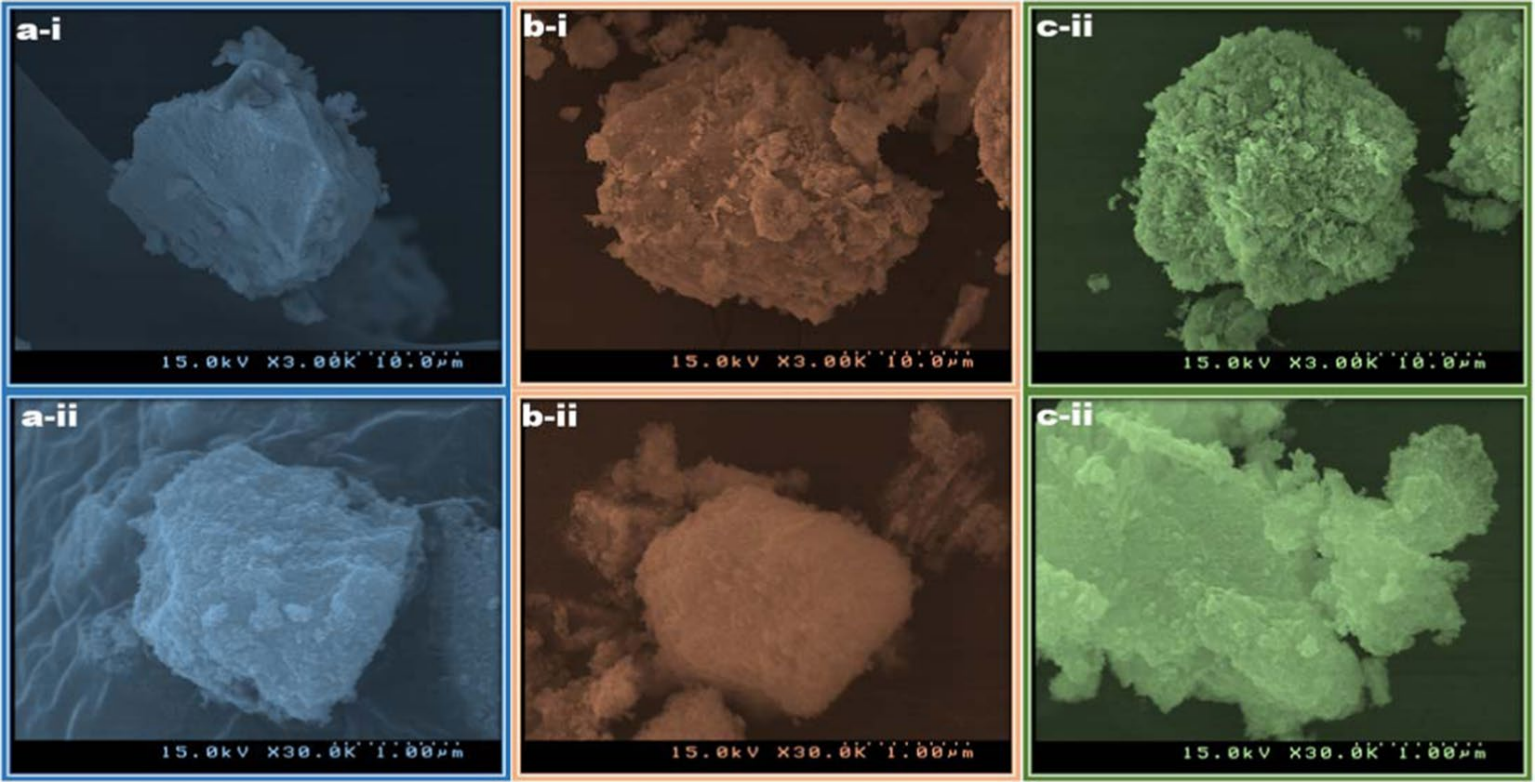
SEM images of the cerium oxide nanocubes as a function of urea concentrations, (**a-i**,**ii**) CeO_2_ NCs-0.5, (**b-i**,**ii**) CeO_2_ NCs-1.0, and (**c-i**,**ii**) CeO_2_ NCs-2.0.

**Figure 6:**
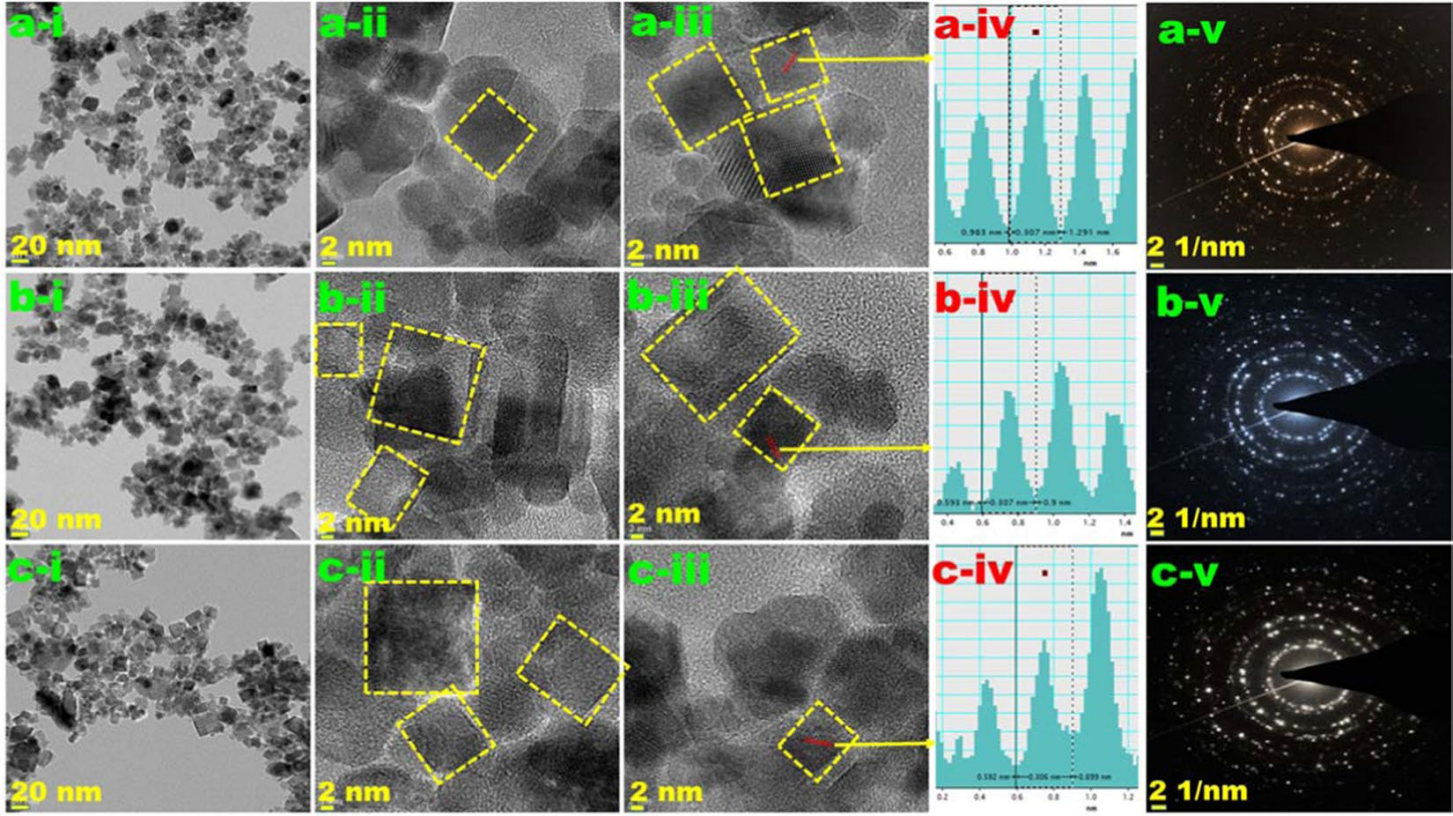
HR-TEM images of the cerium oxide nanocubes as a function of urea concentrations. The (**a**) top, (**b**) middle, and (**c**) bottom panels correspond to CeO_2_ NCs-0.5, CeO_2_ NCs-1.0, and CeO_2_ NCs-2.0, respectively. The panels labeled i–iii show different magnifications. The respective panels labeled iv shows the *d*-spacing values and line profiles, and v correspond to the SAED patterns.

The morphology of CeO_2_ NCs was obtained by SEM, and the images are revealed in Fig. 5. For CeO_2_ NCs-0.5, CeO_2_ NCs-1.0, and CeO_2_ NCs-2.0, SEM images show that aggregates were formed by the accumulation of nanocubes. SEM images of CeO_2_ NCs-0.0 was shown in Fig. S3, the results are indicating that the CeO_2_ NCs-0.0 were more in aggregation and size also higher than the urea mediated CeO_2_ NCs. The details of CeO_2_ NCs were further confirmed by HR-TEM studies. Fig. 6 shows the HR-TEM images of CeO_2_ NCs synthesized with different concentrations of urea. The TEM images show that CeO_2_ NCs were well dispersed, almost polydispersed, consisting of cubic shapes. The CeO_2_ NCs obtained with 0.5 urea-based NCs were smaller in size and less aggregated compared with the 1.0 and 2.0 urea-based NCs. To confirm the phase formation of the NCs, HR-TEM images were analyzed using the Gatan software, and the results are shown in Fig. 6a-iv–c-iv for CeO_2_ NCs-0.5, CeO_2_ NCs-1.0, and CeO_2_ NCs-2.0, respectively. The lattice fringe distance values were similar, 0.307 nm for CeO_2_ NCs-0.5, 0.307 nm for CeO_2_ NCs-1.0 and 0.306 nm CeO_2_ NCs-2.0, and were assigned to the (111) plane of the CeO_2_ NCs. The corresponding selected area electron diffraction (SAED) pattern (Fig. 6a–v, b–v and c–v for CeO_2_ NCs-0.5, CeO_2_ NCs-1.0, and CeO_2_ NCs-2.0) shows well-defined diffraction rings that confirm the polycrystalline nature of CeO_2_ NCs. These results were in good agreement with the XRD results. Therefore, the HR-TEM images revealed a good crystalline nature of the urea assisted NCs.

### Catalytic activity

**Figure 7:**
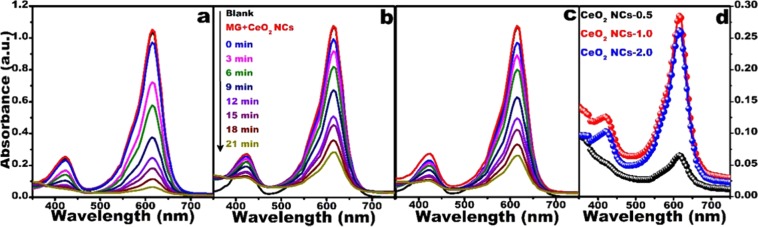
Time-dependent UV-visible absorption spectra of MG by NaBH_4_ in the presence of (**a**) CeO_2_ NCs-0.5, (**b**) CeO_2_ NCs-1.0, and (**c**) CeO_2_ NCs-2.0, and (**d**) comparison of catalytic activity of the samples at 21 min.

**Figure 8:**
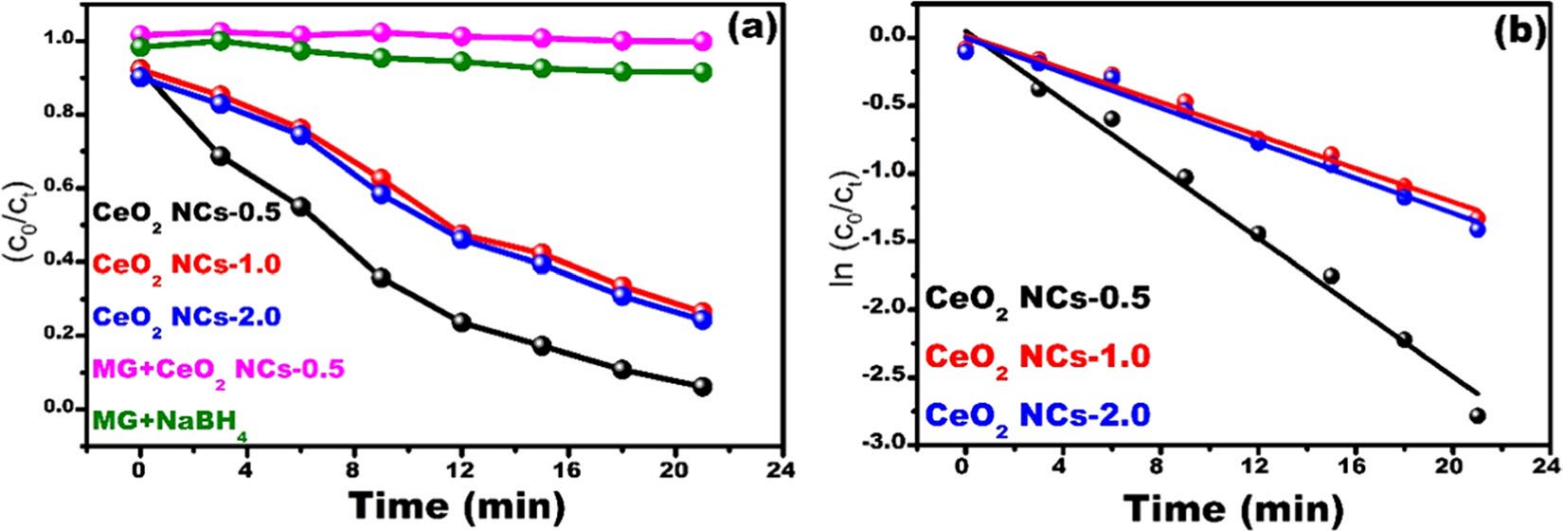
Catalytic activity (**a**), and plot of ln (C_0_⁄Ct) (**b**) of CeO_2_ NCs as a function of urea concentrations.

MG dye was selected as a model pollutant to examine the catalytic activity of CeO_2_ NCs in the presence of NaBH_4_ as the reducing agent under ambient conditions. The results are presented in Fig. 7a to c for CeO_2_ NCs-0.5, CeO_2_ NCs-1.0, and CeO_2_ NCs-2.0, respectively. Fig. 7d shows a comparison of the absorption spectra of the catalytically degraded MG after 21 min in the presences of catalyst and NaBH_4_. The MG maximum absorption peak intensity was at~617 nm. The intense green color of the MG dye solution slowly faded becoming colorless, during the catalytic dye degradation process. A plot of *C*_*o*_/*C*_*t*_ versus degradation, time is shown in Fig. 8a for all samples, where *C*_*o*_ is initial concentration and *C*_*t*_ is concentration at time ‘t’.

Langmuir-Hinshelwood pseudo-first order kinetics was applied to determine the first-order rate constant for dye degradation using the following relation^68^:

5 $${\rm{k}}=-\,ln({C}_{o}/{C}_{t})$$

where ‘k’ is the rate constant (min^−1^) of the dye degradation reaction, *C*_*o*_ is the initial concentration and *C*_*t*_ is the concentration of the dye solution after time ‘t’ in minutes, respectively. The results are shown in Fig. 8b. To identify the role of catalyst, we performed two control experiments. In the first experiment we performed the reaction between NaBH_4_ + CeO_2_ NCs and in the second experiments, we performed the reaction between dye (MG) + NaBH_4_, respectively. In both these control experiments, there was no observed degradation, based on these control experimental results we confirmed that the catalyst (CeO_2_ NCs) playing a vital role in the dye decolorization, and when both the catalyst + NaBH_4_ were together, the degradation materialized rapidly; the results are shown in Fig. 7a. The CeO_2_ NCs-0.5 exhibited an excellent dye degradation ~90%, while the CeO_2_ NCs-1.0 and CeO_2_ NCs-2.0 exhibited ~70% MG dye degradation within 21 min. The possible mechanism of dye reduction by the catalyst is explained by the electron relay system. The NCs start the catalytic reduction by relaying electrons from the donor $B{H}_{4}^{-}$ to the dye molecules (acceptor), where the catalyst (NCs) accepted electrons from nucleophilic $B{H}_{4}^{-}$ ions and transferred them to electrophilic dyes. Saikia *et al*. described hydrogen species generated from $B{H}_{4}^{-}$ ions attack the dye (MG) molecules after electron transfer to nanocubes^26^. This reaction leads to a reduction in the MG dye, which is generally leuco MG (colorless). The CeO_2_ nanocatalysts show slope of −0.127, −0.061 and −0.064 for CeO_2_ NCs-0.5, CeO_2_ NCs-1.0, and CeO_2_ NCs-2.0, respectively. The rate constants for dye degradation were 0.127 min^−1^, 0.061 min^−1^, and 0.064 min^−1^ for CeO_2_ NCs-0.5, CeO_2_ NCs-1.0, and CeO_2_ NCs-2.0, respectively (Fig. 8a,b). CeO_2_ NCs-0.5 exhibited the highest dye degradation due to their size and high dispersion. We evaluated the catalytic activity of the CeO_2_ NCs-0.0, the rate constant was 0.03 min^−1^, as shown in Fig. S3 and it has shown poor performance than CeO_2_ NCs-1.0 and 2.0 that of counterparts due to bigger size and aggregation of particles.

## Conclusion

We developed cost-effective CeO_2_ NCs using carbamide by one-pot hydrothermal method. The HR-TEM images revealed the synthesized NCs was cubic in shape. XRD results show the synthesized nanoparticles MGS of 12, 12, 13 and 31 nm for CeO_2_ NCs-0.5, CeO_2_ NCs-1.0, CeO_2_ NCs-2.0 and CeO_2_ NCs-0.0, respectively. These results were indicating that urea acting as capping and reducing agent. The synthesized CeO_2_ NCs also acted as efficient catalysts in the degradation of the MG dye.

## Supplementary information

Supplementary Information
