# Supplementary material for: Urea assisted ceria nanocubes for efficient removal of malachite green organic dye from aqueous system
Source: Sci Rep. 2019 Oct 9;9:14477. doi: 10.1038/s41598-019-50984-6 (PMC6785541; doi:10.1038/s41598-019-50984-6)
Supplement: Supplementary file 1 — Supplementary Information [file 41598_2019_50984_MOESM1_ESM.docx]

**Supplementary information**

**Urea assisted ceria nanocubes for efficient removal of malachite green organic dye from aqueous system.**

Thupakula Venkata Madhukar Sreekanth^1^, Patnamsetty Chidanandha Nagajyothi^1^, Gutturu Rajasekhara Reddy^2^, Jaesool Shim^1,^ and KisooYoo^1*^

^1^ School of Mechanical Engineering, Yeungnam University, Gyeongsan-38541,

Republic of Korea

^2^ Department of Instrumentation, Sri Venkateswara University, Tirupati 518 502, India

*** Corresponding author:** Prof. Kisoo Yoo, Email: [kisooyoo@yu.ac.kr](mailto:kisooyoo@yu.ac.kr)


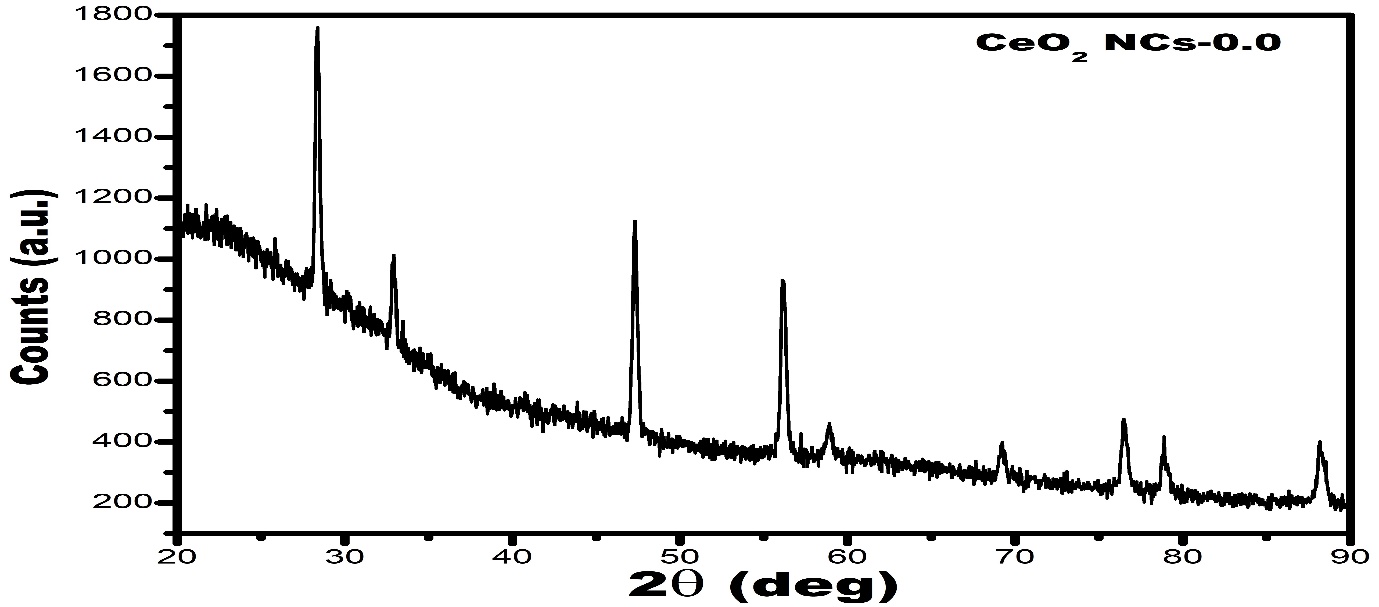


**Figure S1.** XRD spectrum of the cerium oxide nanocubes (CeO_2_ NCs-0.0) without urea.


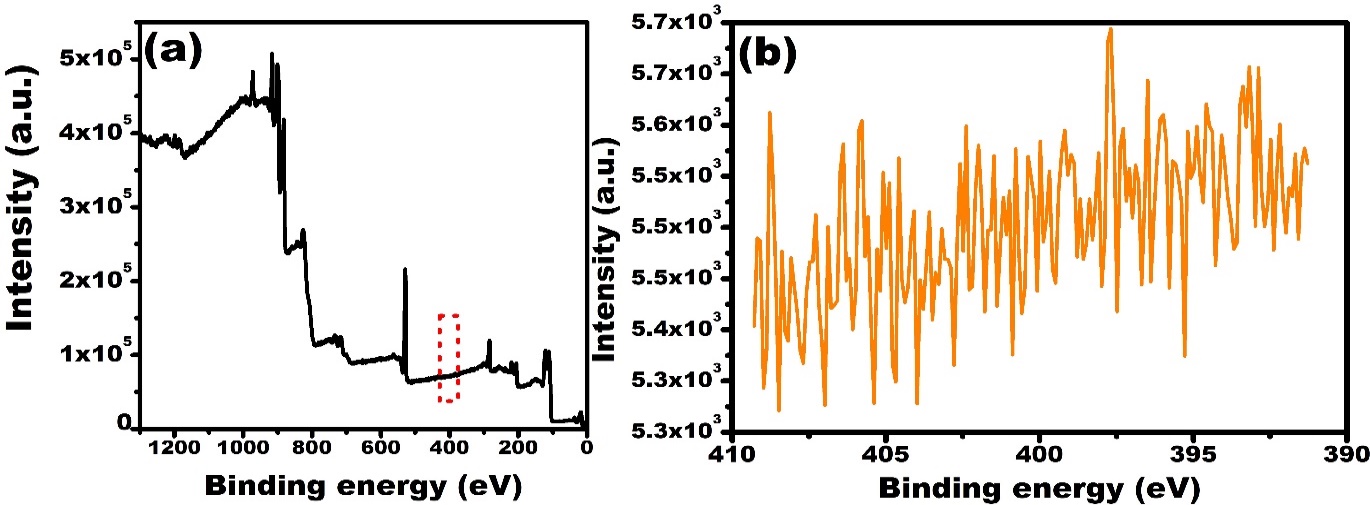


**Figure S2.** XPS analysis of cerium oxide nanocubes (CeO_2_ NCs-1.0), survey scan (a) and high-resolution scan of N 1s (b).


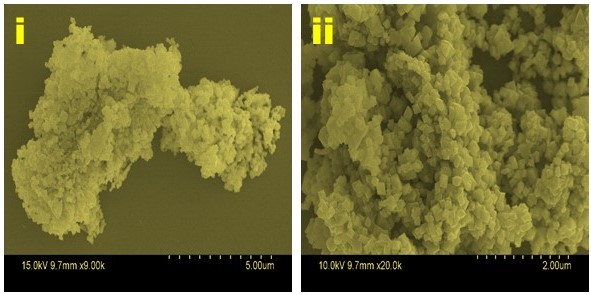


**Figure S3.** SEM images of cerium oxide nanocubes (CeO_2_ NCs-0.0), without urea.


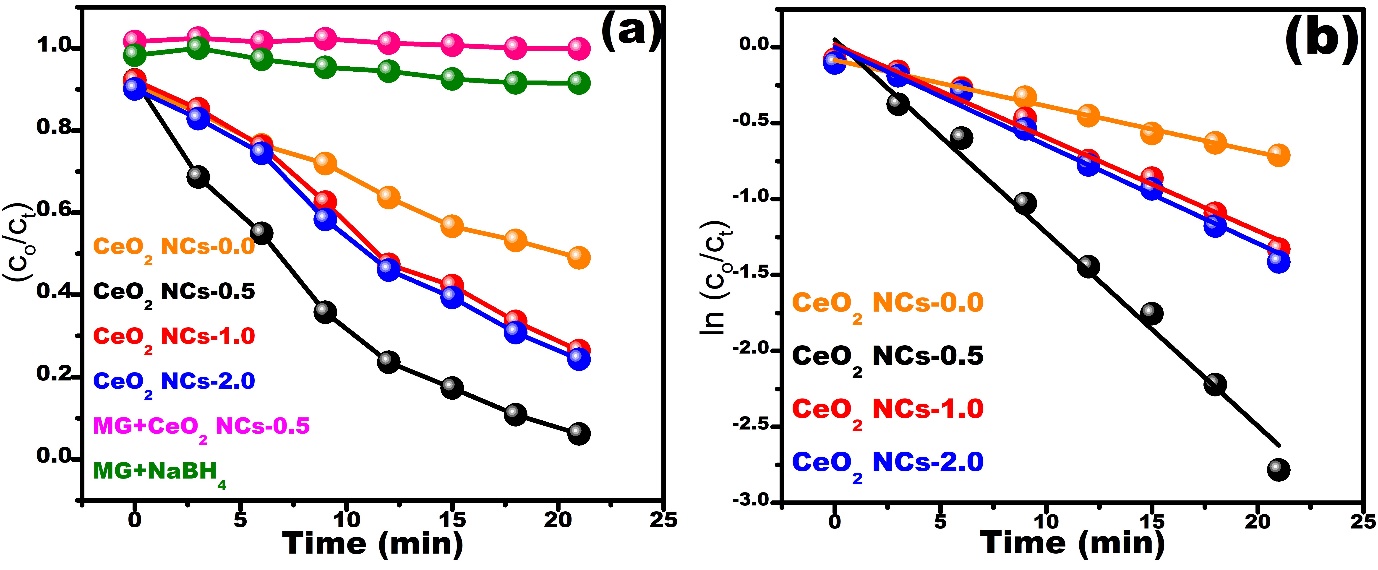


**Figure S4.** Catalytic activity (a), and plot of $ln \left( {C_{o}}/{C_{t}} \right)$ of CeO_2_ NCs with and without urea (b).
